# Modulation of the Mas-Related G Protein-Coupled Receptor X2 (MRGPRX2) by Xenobiotic Compounds and Its Relevance to Human Diseases

**DOI:** 10.3390/jox14010024

**Published:** 2024-03-13

**Authors:** Alicja Dziadowiec, Iwona Popiolek, Mateusz Kwitniewski, Grzegorz Porebski

**Affiliations:** 1 Department of Clinical and Environmental Allergology, Jagiellonian University Medical College, 31-503 Krakow, Poland; alicja.dziadowiec@doctoral.uj.edu.pl; 2 Department of Immunology, Faculty of Biochemistry, Biophysics and Biotechnology, Jagiellonian University, 30-387 Krakow, Poland; mateusz.kwitniewski@uj.edu.pl; 3 Department of Toxicology and Environmental Diseases, Jagiellonian University Medical College, 31-503 Krakow, Poland

**Keywords:** xenobiotic, MRGPRX2, mast cell, polyphenols, flavonoids, coumarins, alkaloids

## Abstract

Mast cells (MCs) are immune cells that reside in tissues; particularly in the skin, and in the gastrointestinal and respiratory tracts. In recent years, there has been considerable interest in the Mas-Related G Protein-Coupled Receptor X2 (MRGPRX2), which is present on the surface of MCs and can be targeted by multiple exogenous and endogenous ligands. It is potentially implicated in non-IgE-mediated pseudoallergic reactions and inflammatory conditions such as asthma or atopic dermatitis. In this paper, we review natural products and herbal medicines that may potentially interact with MRGPRX2. They mainly belong to the classes of polyphenols, flavonoids, coumarins, and alkaloids. Representative compounds include rosmarinic acid, liquiritin from licorice extract, osthole, and sinomenine, respectively. While evidence-based medicine studies are still required, these compounds have shown diverse effects, such as antioxidant, analgesic, anti-inflammatory, or neuroprotective. However, despite potential beneficial effects, their use is also burdened with risks of fatal reactions such as anaphylaxis. The role of MRGPRX2 in these reactions is a subject of debate. This review explores the literature on xenobiotic compounds from herbal medicines that have been shown to act as MRGPRX2 ligands, and their potential clinical significance.

## 1. Introduction

Mast cells (MCs) are, among a number of other functions, the primary initiators of allergic and allergic-like symptoms; they swiftly release numerous mediators upon activation. Allergic MC activation occurs via an IgE-dependent pathway, in which the allergen is matched to a specific IgE that binds to a high-affinity IgE receptor (FcεRI) on the cell surface [1]. However, there are also clinical reactions that resemble allergy and develop after exposure to a variety of xenobiotic compounds for which IgE-mediated mechanisms have not been demonstrated and are therefore termed pseudoallergic or anaphylactoid [2]. After a period of uncertainty regarding the responsible pathway, the Mas-Related G Protein-Coupled Receptor X2 (MRGPRX2) was proposed to be one of the possible IgE-independent MC activation pathways [3]. McNeil et al. demonstrated that MRGPRX2 can be activated by xenobiotics, including fluoroquinolones, neuromuscular blocking agents, and peptidergic therapeutics (e.g., icatibant, leuprolide), in addition to previously known endogenous ligands such as neuropeptides and substance P (SP) [4]. Since the publication of McNeil’s seminal paper in 2015, the number of publications addressing xenobiotic triggering of MRGPRX2 has increased rapidly. The hypothesis that drug hypersensitivity reactions are induced via an MRGPRX2-dependent pathway, mainly by drugs from the muscle relaxant and flouroquinolone antibiotic groups, has attracted much attention from the scientific community [5]. However, many studies have also been devoted to other xenobiotics—including those found in medicinal plants—and these analyse their association with the MRGPRX2 receptor. In this article, we review these studies, focusing on the best documented evidence and the most representative compounds derived from the polyphenols, coumarins, alkaloids, and other groups that could have a potential impact on accelerating or alleviating MRGPRX2-dependent diseases. In addition to an analysis of the latest available data on the association of MRGPRX2 with xenobiotics and its potential clinical relevance, we also discuss the limitations of these studies, highlighting the current knowledge gap.

## 2. Pathophysiological Basis

### 2.1. Mast Cell Characteristics

MCs are immune cells that are present in almost all tissues of the body but are particularly abundant in those tissues directly exposed to the external environment [6]. While MCs are primarily associated with allergic reactions, they also play a significant role in various physiological and pathological processes [7,8,9,10,11].

All MCs contain intracellular granules and express the high-affinity IgE receptor FcεRI on their surface [12]. The cross-linking of FcεRI receptors upon antigen-IgE binding is the most recognized pathway of MC activation, playing a crucial role in potentially fatal reactions such as anaphylaxis [1]. MC stimulation leads to degranulation and the release of granule contents, which is a primary cause of hypersensitivity manifestations [1]. The granules store a wide range of preformed mediators, including histamine [13], proteases such as tryptases and chymases [13,14], and also some cytokines; mainly tumor necrosis factor alpha (TNF-α) [15]. These substances cause various biological effects, such as increasing vascular permeability, smooth muscle contraction and activation of immune cells, which are associated with symptoms of allergic inflammation [16]. In addition to the immediate release of preformed mediators, MCs also secrete de novo synthesized compounds that are produced after MC stimulation [13]. These include lipid mediators—such as prostaglandin D2 (PGD2), which are rapidly produced and released [17]—and cytokines, which are produced and secreted over a longer period of time (hours rather than minutes) [18,19,20].

In humans, MCs are generally categorized into one of three subtypes, based on the content of specific proteases. MCs that contain only tryptase (MC_T_) are found in the mucosa of the small intestine and in the alveolar septa [21]. MCs that contain only chymase (MC_C_) are commonly found in synovial tissue. MCs, which contain both tryptase and chymase (MC_TC_), are predominantly found in the skin, submucosal layers of the small intestine, and tonsils [22]. However, at the transcriptional level, the protease content displays more tissue-specific variability, which is evident both between and within tissues [12]. Cutting-edge advancements in single-cell profiling technologies have opened new avenues to unravel the complexity and diversity of MCs. These breakthroughs shed light on previously unseen heterogeneity among MCs across various tissues, which is distinct from other cell types. In humans, transcriptomic analysis unveiled the existence of seven distinct MC subsets (MC1–7) distributed across 12 organs, each with unique transcriptomic core signatures [23].

All MCs express FcεRI, but there is controversy regarding whether MC_T_ and MC_C_ express MRGPRX2, despite the known expression of MRGPRX2 in skin MC_TC_ [24,25,26]. Furthermore, even among skin MC_TC_, only a small percentage of cells exhibit MRGPRX2 expression under steady-state conditions [24,25].

### 2.2. Structure and Regulation of MRGPRX2 Function

**Figure 1:**
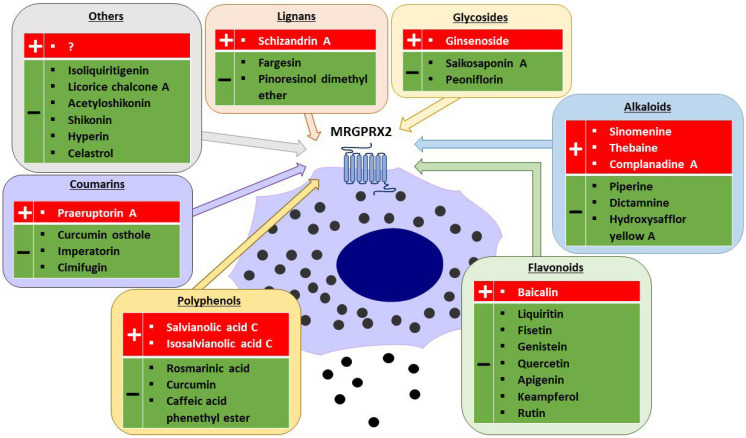
Plant-derived agonists (+) and inhibitors (−) of MRGPRX2 (created with Motifolio, Motifolio Inc., Elliocott City, MD, USA).

MRGPRX2 is a G protein-coupled receptor (GPCR) that was first reported to be expressed mainly on MCs and sensory neurons [3,27]. The receptor has low affinity and low selectivity with respect to ligand binding. MRGPRX2 has been shown to be activated by a wide range of endogenous and exogenous compounds, primarily by small cationic molecules and peptides that have amphipathic properties, or share a motif of tetrahydroisoquinoline (THIQ) or a similar motif [4,5]. Endogenous ligands of MRGPRX2 include neuropeptides such as SP, PAMP-12, and cortistatin-14 (CST-14), as well as antimicrobial host defense peptides such as cathelicidin LL-37, hBD2, and eosinophil granule proteins (e.g., MBP). Exogenous ligands of MRGPRX2 include the cationic polymer compound 48/80 (C48/80), which is commonly used in receptor functional assays, and a variety of drugs approved by the Food and Drug Administration (FDA), such as fluoroquinolones (e.g., ciprofloxacin), neuromuscular blocking agents (e.g., rocuronium, atracurium), opioids (e.g., morphine), and many others [4,9,28]. MRGPRX2 can also be activated or inhibited by other exogenous agents, such as bacterial quorum sensing proteins, insect venoms [3,29,30], or many different plant xenobiotics (Figure 1) [31,32,33,34,35,36,37,38,39,40,41,42,43,44,45,46,47,48,49,50,51,52,53,54,55,56,57,58,59]; representatives of which are described in the following part of this review.

As a GPCR, MRGPRX2 shares the structure of seven transmembrane (TM) α-helices connected by three extracellular loops (ECLs) and three intracellular loops (ICLs) [60]. The ECL region contains the N-terminus responsible for ligand binding, whereas the ICL region contains the C-terminus involved in G protein coupling, β-arrestin recruitment, and downstream signalling [61,62,63,64]. The extracellular binding of ligands to MRGPRX2 promotes the conformational changes in the transmembrane domains, resulting in structural changes on the cytoplasmic side of the membrane and activation of G proteins, and subsequent MC degranulation [65]. Conversely, some ligands can induce intracellular β-arrestin recruitment, leading to receptor desensitization and internalization [62]. The downstream signalling pathways of MRGPRX2 involve the activation of the phospholipase C pathway (PLC-PKC-IP3R), which result in intracellular Ca^2+^ influx and MC degranulation. Additionally, the MAP kinase (ERK-P38-JNK), PI3K-AKT, and NF-κB pathways are activated, leading to cytokines and PGD2 synthesis in MCs [7].

### 2.3. Role of MRGPRX2 in MC-Driven Skin Diseases

To date, the exact role of MRGPRX2 in MCs has not been fully understood [9]. Numerous in vivo and in vitro studies have been conducted on the receptor (and its mouse ortholog, MrgprB2 [4]), indicating its potential involvement in various physiological and pathological processes. With its ability to bind to a diverse range of ligands, MRGPRX2 has been implicated in drug pseudoallergic reactions, neurogenic inflammation, and a wide array of inflammatory diseases such as allergic contact dermatitis (ACD), chronic urticaria (CU), rosacea, rheumatoid arthritis, atopic dermatitis (AD), mastocytosis, ulcerative colitis, and allergic asthma [8,9,10,11]. However, conclusive evidence regarding MRGPRX2′s involvement in these conditions in humans is still lacking.

Endogenous peptides considered to be MRGPRX2 ligand play an important role in the development of inflammatory skin diseases. The neuropeptide SP and the host defense peptide cathelicidin LL-37 are key players in the pathogenesis of rosacea and AD, and are upregulated in the skin of patients [8,10]. Both peptides in vitro were shown to activate MCs via MRGPRX2, leading to MC degranulation and release of pro-inflammatory mediators, including histamine and cytokines (i.e., TNFα) [66,67]. It was proposed that the released mediators can subsequently act on sensory neurons and vascular endothelial cells to promote neurogenic inflammation, resulting in itching, erythema, swelling, and pain that exacerbate disease symptoms [8,10]. In addition, MC-derived mediators recruit immune cells into the inflamed tissue and stimulate both neurons and immune cells (such as neutrophils) to secrete more SP and LL-37, which then could again activate MCs [8,10,11]. Similar mechanisms involving SP and MCs are also present in ACD and CU [8,10,68]. Another neuropeptide ligand of MRGPRX2 involved in the development of neurogenic skin inflammation, such as the non-histaminergic pruritus associated with ACD, is CST-14 [8,10,69,70,71]. The skin conditions are also characterized by elevated levels of proinflammatory cytokines such as IL-13 and IL-31 [72,73,74]. It is noteworthy that in all these diseases, except CU, an increased number of MCs has been reported in the skin of patients compared to healthy controls [8,10]. Additionally, the expression of MRGPRX on cutaneous MCs is higher in patients with CU [24]. Therefore, the involvement of MRGPRX2 in inflammatory skin diseases is suggested [8,10,11].

In several of these diseases, the usual treatment with antihistamines and other first-line drugs has been reported to be ineffective [75,76,77]. With the current generation of H1-antihistamines, sedation has become a minor concern, as the use up to fourfold normal doses are minimally or non-sedating [77,78,79]. However, due to incomplete efficacy in all patients, the search for other medications remains a priority.

## 3. Traditional Chinese Medicines and Plant-Derived Compounds

Traditional Chinese medicine (TCM) is a medical therapy system that has been practiced for millennia. It stands as one of the earliest forms of medical practice recorded in global history. Given their extensive use in China and many other countries, traditional Chinese medicines (TCMs) remain among the most commonly prescribed therapeutic agents worldwide [80]. TCMs often consist of natural herbal and other remedies tailored for specific conditions such as allergic or heart diseases [81,82]. Both the beneficial effects and side effects of TCMs may potentially be linked, at least in part, to the interaction of active compounds with the MRGPRX2 receptor.

### 3.1. TCM Compounds in Evidence-Based Medicine and Their Potential for Use in Humans

Evidence-based medicine (EBM) involves making medical management decisions, particularly therapeutic decisions, based on current scientific evidence that has been systematically and reliably verified [83]. EBM is widely accepted in modern medicine in most countries and is based on the results of high-quality clinical trials; typically randomized, double-blinded, and placebo-controlled trial. In contrast, evidence from case-control studies, followed by case reports or studies in animal models or in vitro, is of less importance [83]. Chinese medicine, including treatment with herbs and their constituents, has a fundamentally different approach to therapeutic decision-making. The approach is based on a long-standing tradition, there is a lack of well-designed, standardized and reproducible clinical trials to demonstrate the efficacy and safety of the therapeutic interventions used [84,85]. TCM studies receive an average low mean score of 1.25 on the Jadad scale which is used to assess the methodological quality of clinical trials; a maximum score of 5 indicates the best-quality study [86]. The quality of clinical trials in TCM is limited by several factors. These include batch-to-batch variation of investigational products, difficulty in preparing appropriate placebos for multicomponent herbal preparations, unclear randomization rules, and the discrepancy between the standardized intervention required by EBM and the individual patient approach inherent in TCM [84]. Therefore, data obtained within the TCM context are major risk factors for bias and may limit the translatability of these findings to an evidence-based clinical context.

Although there is increasing evidence of the biological activity of many xenobiotic compounds used in TCM formulations, this evidence is mostly derived from animal or in vitro models that evaluate the effects of specific isolated compounds at concentrations that may not be biologically relevant or representative of therapeutically used extracts (refer to Table 1). For instance, baicalin was claimed to possess “anticancer” activities in non-small cell lung cancer, but these conclusions came from a study that assessed its effect on tumor growth and survival in a mouse model [87]. The design of this study suggests that “anticancer” property should be considered as antineoplastic activity (i.e., elimination of cancer cells). In another example concerning cancer the authors showed, through experiments on cell culture, that osthole inhibits proliferation of gastric cancer cells [88]. Molecular modelling methods were applied in another study to target signalling pathways involved in breast cancer development with molecules such as salvianolic acid C [89]. This is also research that is still distant from clinical applications. It should therefore be noted that often the properties of TCM substances refers to their effects in experiments conducted, as mentioned above, in vitro and such properties cannot be directly translated into the clinic until they have been proven though robust placebo-controlled trials. It should also be emphasized that a number of the substances described below have not been registered as medicinal products by the FDA and the European Medicines Agency [90,91]. In TCM, these substances are attributed with anti-inflammatory effects (osthole [92,93,94,95,96,97], flavonoids [98,99,100]); have been proposed for use in heart disease (salvianolic acid C [101,102]), rheumatoid arthritis (sinomenine [103]), cardiovascular disease, gastrointestinal and respiratory infections (baicalin [104]); however, they also have very broad and non-specific indications (liquiritin [105], praeruptorin A [106], piperine [107], rosmarinic acid [108]).

Clinical data on interventions based on MRGPRX2 inhibition are limited. However, it is worth noting that in-human studies are already underway. Following successful basic in vivo studies in mouse and dog models [109], two clinical trials have been initiated with an orally administered specific MRGPRX2 antagonist—the synthetic small molecule compound EP262—in the indications of chronic spontaneous urticaria and atopic dermatitis [110]. Both studies are double-blinded, placebo-controlled, and randomized; therefore, they are expected to provide reliable results on the efficacy of the studied molecule. The primary outcome measure for urticaria is the change in a patient-reported questionnaire assessing the number of hives and intensity of itch over seven consecutive days. In the atopic dermatitis study, the primary objective is to evaluate the safety and tolerability of EP262. The results of these studies are expected to provide valuable insight into the practical clinical relevance of blocking MCs degranulation in the MRGPRX2-dependent pathway.

### 3.2. Polyphenols

Polyphenols are a broad and complex category of chemical substances derived from plants. These components have at least one aromatic ring with one or more hydroxyl (OH) groups in their structure, and are categorized into several classes; of which flavonoids, phenolic acids, lignans, and stilbenes are the main groups [111]. Polyphenols are commonly found in fruits and vegetables [111]. Few randomized clinical trials have demonstrated antioxidant, antidiabetic, and cardioprotective activity of some polyphenols, or their role in improvement of gut microbial composition as prebiotics [112,113,114,115,116,117,118,119]. Additionally, these are suggested to display many other biological effects such as anti-aging, anti-inflammatory, anticarcinogenic, and neuroprotective. However, direct in-human evidence on these alleged properties are so far lacking [111]. Several polyphenols have been reported as potential MRGPRX2 ligands exerting possible protective or pathological effects in chronic skin diseases and other conditions, including MRGPRX2-dependent pseudoallergy reactions [33,35,37,120].

#### 3.2.1. Salvanolic Acid C and Isosalvanolic Acid C

**Figure 2:**
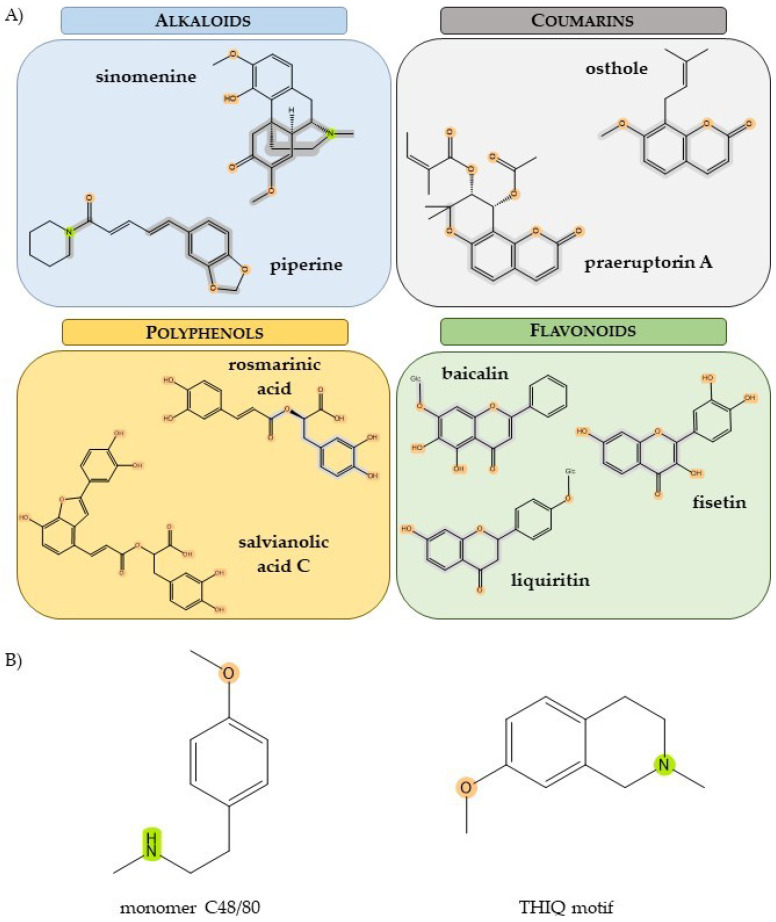
(**A**) Structure diagrams of selected compounds in the different ligand groups. (**B**) Two potent mast cell degranulators: monomer C48/80 and tetrahydroisoquinoline (THIQ) motif. Molecular fragments with patterns similar to monomer of C48/80 as well as heterocyclic motifs from THIQ are highlighted (created with ChemOffice 22.0, Perkin Elmer, Shelton, CT, USA).

Danshen injection (DI) is a traditional Chinese medicine injection solution (TCMI), containing the primary component derived from *Salvia miltiorrhi* [37,120]. It is commonly used in the medical treatment of angina pectoris [102], liver cirrhosis [121], and heart diseases including acute coronary syndrome [122]. However, the use of DI is often associated with adverse reactions, including anaphylaxis [123,124]. Three phenolic acids of *Salvia miltiorrhi*—namely salvianolic acid A (SA), salvianolic acid C (SC), and isosalvianolic acid C (ISC) (Figure 2)—have been identified as MRGPRX2 agonists and have been shown to induce degranulation of MCs [37,120]. Among them, SC was shown to exhibit the most potent MC stimulating activity. In the intracellular Ca^2+^ mobilization assay on MRGPRX2-transfected human embryonic kidney 293 (MRGPRX2-HEK293) cells, a half maximal effective concentration (EC_50_) of the components was determined. The EC_50_ of SC, ISC and SA were 15.70 ± 4.62, 38.88 ± 8.67, and 363.40 ± 34.51 μM, respectively [37]. These results were confirmed by cell membrane chromatography, which showed that SC had the longest retention time on the column with MRGPRX2, indicating the strongest interaction with the receptor [37]. The authors also suggested that these polyphenolic compounds compete to bind to the active site of MRGPRX2 with ciprofloxacin, which is a known receptor ligand [37]. Molecular docking of ISC subsequently supported this hypothesis, showing that ISC forms at least three hydrogen bonds with MRGPRX2 in the active pocket [120]. In a mouse model of passive cutaneous anaphylaxis (PCA), the injection of SC and ISC into the mouse hind paw resulted in tissue swelling and increase of vascular permeability [37]; whereas hind paw inflammation was significantly inhibited in MrgprB2 knockout mice or mice with MCs depletion [120]. Furthermore, the activation and degranulation of Laboratory of Allergic Disease 2 (LAD2) human mast cells induced by SC and ISC was abolished in MRGPRX2 knockout cells [37,120]. These reports suggest that the polyphenolic compounds found in DI may be responsible for anaphylactoid reactions to the drug, especially two geometric isomers, SC and ISC. It is noteworthy that DI research has demonstrated the instability of SA in distilled water solutions, resulting in its conversion to SC and ISC, which are the more potent components [125]. However, the complex composition of DI does not exclude the involvement of other substances in the induction of the anaphylactoid reactions [124,126]. Similarly, the administration route, including the high dose of DI, which was described as the cause of some adverse drug reactions (ADRs) [127,128], could be the reason for allergy-like reactions to DI. The data highlight the need for caution in the administration of TCMI and the urgent need for in-depth research of TCMs ingredients.

#### 3.2.2. Rosmarinic Acid

**Figure 3:**
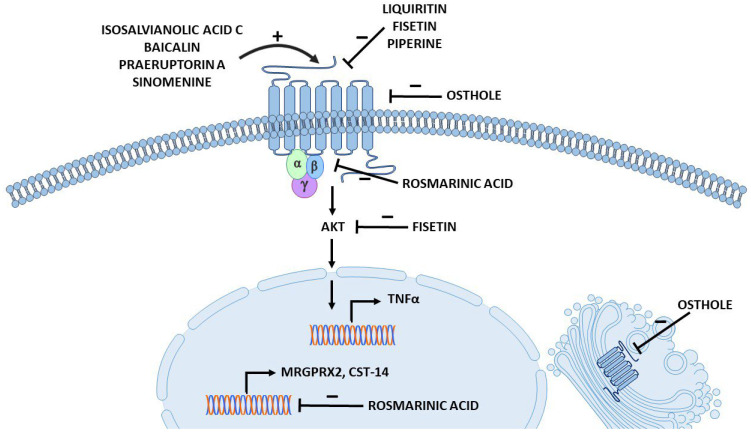
Proposed action points for different compounds affecting MRGPRX2 (created with Motifolio Inc., Elliocott City, MD, USA).

Rosmarinic acid (Figure 2) is a polyphenolic compound commonly found in *Rosemarinus officinalis*, popularly known as rosemary, and in other herbs, such as *Perilla frutescens*, which is widely used in TCM [108]. In vitro studies and animal models suggest that rosmarinic acid can have some physiological effects, including anti-inflammatory and antinociceptive activities (Table 1) [129,130]. One of its proposed mechanisms of action is the targeting of signalling pathway proteins, such as NF-κB [131]. Recently, data have emerged suggesting an ameliorative effect of rosmarinic acid on ACD and inhibition of MRGPRX2-mediated pseudoallergic reactions [35,72]. Ding et al. established a mouse model of dibutyl square acid-induced ACD that exhibits common symptoms of ACD, such as epidermal thickness, lymphocyte infiltration, MC degranulation, elevated serum levels of histamine and IL-13, and increased bouts of scratching in mice [72]. Notably, rosmarinic acid treatment reduced all symptoms of ACD in the mouse model. Furthermore, the ACD model exhibited increased expression of CST-14 mRNA, which was significantly decreased after administration of rosmarinic acid. The role of rosmarinic acid in MRGPRX2-mediated MC activation was also investigated. The results showed that pretreatment with rosmarinic acid reduced intracellular Ca^2+^ influx, LAD2 cell degranulation, and histamine release induced by CST-14 and C48/80 [35,72]. Furthermore, in cells treated with rosmarinic acid, MRGPRX2 mRNA and protein levels were downregulated whereas CST-14 expression levels remained unchanged [72]. The suppressing effect of rosmarinic acid on MC stimulation was also demonstrated to inhibit the activation of downstream signalling pathways. The compound decreased the phosphorylation of proteins associated with MC degranulation (PLC and IP3) and cytokine production (PKC and ERK), as well as NF-κB; this is consistent with previous studies [131]. Interestingly, the authors also performed a molecular docking study, in which they demonstrated that rosmarinic acid interacts with MRGPRX2 and associates with its G proteins at the intracellular site (Figure 3) [72]. Conversely, another study [40] indicated that rosmarinic acid is a weak inhibitor (IC_50_ = 1.8 mM) of C48/80-induced activation of MCs. However, the study was conducted in other cell lines, namely primary cell culture of mouse peritoneal mast cells (MPMC) and MRGPRX2-HEK293 cells, and the concentration of rosmarinic acid used in the study were significantly lower (10 µM versus 25–100 µM in the previous studies), which could have a big impact on data results. Additionally, the study did not provide additional evidence supporting weak performance of rosmarinic acid on MC activation [40]. On the other hand, the authors also performed molecular docking and showed that rosmarinic acid was not expected to interact with the MRGPRX2 binding pocket [40]. In conclusion, additional studies should be conducted to elucidate the effect of rosmarinic acid on MRGPRX2-mediated MC functions.

### 3.3. Flavonoids

Flavonoids are a large group of plant compounds—i.e., a subgroup of polyphenols—with a wide range of beneficial effects on human health [98]. They are very abundant in plants, including fruits and seeds; and contribute to their characteristics such as color, fragrance, and flavor [98]. Flavonoids have been reported to potentially exert biological activities such as anti-inflammatory, immunomodulatory, antibacterial, antiparasitic, antiviral, anticancer, anti-aging, neuroprotective, cardioprotective, and antidiabetic effects [98]. Several flavonoids have been identified as potential MRGPRX2 antagonists (Figure 1). Most of them have been reported to have protective effects against hypersensitivity reactions and other health conditions such as pruritus [56] or CU [42,132]. Additionally, agonists of MRGPRX2 among flavonoids have also been identified [33,133]. Flavonoids have been reported to affect MC stimulation both by direct binding to the receptor and by interactions with regulatory enzymes or transcription factors [42,132,134,135]. In this section, we describe a few representatives of flavonoids that may interact directly with MRGPRX2.

#### 3.3.1. Baicalin

Baicalin, a flavone (Figure 2), is one of the major components of *Scutellaria baicalensis* Georgi, which is extracted from a dried root of the plant [33]. Baicalin is commonly used in TCMI for the treatment of inflammation, cardiovascular disease, and gastrointestinal and respiratory infections [104]. Although baicalin has multiple beneficial pharmacological activities (Table 1), TCMI with baicalin as the main active ingredient have been reported to induce a number of allergic reactions [67,136,137,138,139]. Therefore, Wang et al. investigated the role of baicalin in anaphylactoid reactions in mice [33]. Using mouse models of systemic and cutaneous anaphylaxis in wild type (WT) and MrgprB2 knockdown mice, the authors showed that baicalin induces receptor-dependent pseudoallergy [33]. Another study [133] demonstrated that this compound induces intracellular Ca^2+^ mobilization and histamine release in LAD2 cells, but not in MRGPRX2 knockdown LAD2 cells. Taken together, these data suggest that baicalin may induce anaphylactoid reactions to TCMI through MRGPRX2.

#### 3.3.2. Liquiritin from Licorice Extract

Licorice (synonyms: liquorice, and Gan-Cao in Chinese [139]) is scientifically known as the genus *Glycyrrhiza* [105] and is widely used in the food industry (as flavoring and sweetener agents [31]), in cosmetics and in pharmaceuticals [105]. At the same time, licorice is one of the most widely used ingredients in TCM [31]. It has many alleged biological effects, including protective activities against many types of cancer [105], antibacterial and anti-inflammatory effects [105]. Licorice extract contains a wide range of bioactive components, including flavonoids such as liquiritin, chalconoids (isoliquiritigenin and licochalcon A), and saponins (glycyrrhizic acid) (Figure 1) [31,41,52,105]. Recent studies have proposed licorice ingredients as treatment agents for MRGPRX2-mediated anaphylactoid reactions [31,41,52]. In vitro studies of one of the active licorice flavonoids, liquiritin (LQ), demonstrated suppression of MC activation (intracellular Ca^2+^ mobilization assay) and degranulation (β-hexosaminidase and histamine release) [41]. The compound also showed an in vivo protective effect against anaphylaxis. In the mouse model of PCA, LQ injection into the hind paw resulted in a dose-dependent suppression of swelling and vasodilation and caused a reduction in the percentage of degranulated skin MCs [41]. The flavonoid also reduced histamine and inflammatory cytokines levels in the paw and serum of mice [41]. Liquiritin has been demonstrated to bind directly to MRGPRX2, and molecular docking studies indicate that it fits well into the active site of the receptor (Figure 3) [41]. At the same time, the compound showed low cytotoxicity in tested cells and no activating effect on MCs [41]. However, it is worth noting that the study did not include MrgprB2 knockout mice or MRGPRX2 silencing, which constitutes a limitation. Nonetheless, these results suggest that LQ may be a potential MRGPRX2 inhibitor and provide a basis for further research.

#### 3.3.3. Fisetin

Another natural flavonoid that possesses a range of potential health-related bioactive properties is fisetin (Figure 2). Fisetin is found in various fruits and vegetables [140,141] and has been proposed to have anti-inflammatory [99] and antiallergic effects (Table 1) [100]. It has been reported to inhibit several signalling pathways in vitro, including PI3K-Akt-mTOR, P38, and NF-κB [142,143], which were associated with an inhibitory effect in human inflammatory skin models [144]. Recent research on the SP and ovalbumin co-stimulated CU mouse model demonstrated protective effects of fisetin against CU [42]. The compound alleviated the symptoms associated with CU in mice and reduced serum levels of inflammatory mediators such as histamine and TNFα, as well as the infiltration of red blood cells into the tissue and degranulation of skin MCs [42]. Additionally, fisetin suppressed local and systemic anaphylactoid reactions in mice [42]. The study revealed that fisetin exerts its inhibitory effects by binding to the active site of MRGPRX2, thus preventing MCs activation [42]. Fisetin also targets the AKT signalling molecule, which is consistent with previous reports on inhibition of signalling pathways (Figure 3) [142,143,144]. In conclusion, fisetin can be considered as a potential MRGPRX2 antagonist in future research.

### 3.4. Coumarins

Coumarins are secondary metabolites that belong to the benzopyrone family and are commonly found in many plants [145]. They were shown to potentially exhibit a range of pharmacological activities, including anti-inflammatory [146,147], antibacterial, antiviral, antifungal [148], anticancer [149], antihypertensive [145], antioxidant [150], and neuroprotective effects [145]. To date, hundreds of coumarins have been identified and described [145,151]. Here, we present representative examples of coumarins that affect the response of MCs via MRGPRX2 signalling.

#### 3.4.1. Praeruptorin A

Praeruptorins are bioactive coumarins extracted from *Peucedanum* species such as *P. praeruptorum*, which are widely used in TCM [106]. Praeruptorins have many beneficial physiological effects in the treatment of upper respiratory tract infections, cardiovascular, immune, and nervous system diseases [106]. One of these biological compounds is praeruptorin A, which exhibits several bioactive properties (Table 1) and has been studied in the context of MC activation via MRGPRX2 [38]. A competitive binding assay showed that this compound competes with ciprofloxacin for binding to MRGPRX2, suggesting that it may interact directly with the receptor [38]. Stimulation of LAD2 cells by praeruptorin A caused β-hexosaminidase and histamine release [38], suggesting that this compound may trigger MRGPRX2-mediated pseudoallergy reactions; however, the data are very limited and thus further studies are required.

#### 3.4.2. Osthole

Osthole is a coumarin extracted from the dried fruits of the *Cnidium monnieri* Cusson plant (Figure 2). It is used in TCM to treat various pathological conditions. Osthole has been considered to possess anti-inflammatory properties [92,93] and has been shown to have protective effects in animal models of allergic asthma [94,95] and AD [97] (Table 1). The study by Callahan et al. [34] demonstrated that osthole attenuated both the early and late phases of MC activation and allergic inflammation in mice in vivo. The compound significantly reduced intracellular Ca^2+^ mobilization and MC degranulation induced by known MRGPRX2 ligands: SP, C48/80, and LL-37. MCs treated with osthole showed a reduction in cytokine release after MC activation and a significant downregulation of kinases phosphorylation in the downstream signalling pathway [34]. Moreover, in the mouse models of PCA and chronic skin rosacea, osthole attenuated the inflammatory response to C48/80 or LL-37 injection, respectively [34]. The compound reduced mRNA levels of MC inflammatory mediators, the percentage of degranulated MCs; and decreased redness, epidermal thickness, and cellular infiltration in the skin of the treated mice cohort [34]. The authors showed that osthole inhibits MC activation through allosteric rather than competitive interactions with MRGPRX2 (Figure 3) [34]. Furthermore, this study showed that osthole affects both the surface and intracellular expression levels of MRGPRX2, providing another possible way to regulate the MRGPRX2 response in allergic reactions and rosacea [34]. However, another study showed that osthole could induce degranulation in rat basophilic leukemia (RBL-2H3) cells, which have rat homologue of MRGPRX2; MrgprB3, and FcεRI [38]. The authors imply the interaction of osthole with IgE receptor, due to results of competitive binding assay with quercetin (used as ligand of FcεRI in the assay). However, in a later study quercetin has been reported to inhibit MRGPRX2 and MrgprB2 by direct binding [46]. Therefore, due to insufficient data, the conclusions should be drawn carefully.

### 3.5. Alkaloids

Alkaloids are plant and animal metabolites that comprise a wide range of compounds that share nitrogen as a characteristic chemical element present in their structures. As a result of their structural diversity, alkaloids have numerous biological properties and are widely used in modern medicine. Illustrative applications of alkaloids in healthcare include chemotherapy (paclitaxel, vinblastine), analgesics (morphine, codeine), treatment of respiratory diseases (codeine, capsaicin), dietary supplements (piperine), and many others [152]. The classification of plant alkaloids is based on their chemical structure, biochemical precursors, and their occurrence in different plant genera. Here, we describe two alkaloids with opposite effects on MRGPRX2-dependent MC activation, namely sinomenine and piperine, which belong to the opium and piperidine alkaloids, respectively [152].

#### 3.5.1. Sinomenine

Natural opium alkaloids (such as codeine, morphine, and its derivatives such as sinomenine, thebaine, pethidine, etc.) have been extensively described as MRGPRX2 agonists [4,32,39,153]. Sinomenine (Figure 2) is extracted from the root of the medicinal plant *Caulis sinomenii* and is a major active component of TCMI, used to treat rheumatoid arthritis [103,154]. Some studies have confirmed the interaction of sinomenine and MRGPRX2 on MC lines, suggesting a possible contribution of MRGPRX2 in sinomenine anaphylactoid reactions [32,39,153,155]. Liu et al. showed that sinomenine increased intracellular Ca^2+^ influx in LAD2 cells and MPMC [153]. However, the response was significantly reduced in MRGPRX2/MrgprB2 silenced cells [153]. Depletion of MRGPRX2 in LAD2 cells was also associated with the absence of sinomenine-induced degranulation as assessed by β-hexosaminidase and histamine release. Treatment of LAD2 cells with sinomenine for 24 h induced a significant upregulation of MC cytokine expression and secretion, as well as MRGPRX2 protein level in the cells and the phosphorylation levels of signalling pathway proteins (PLC, IP3R, P38, PKC). These responses were significantly reduced in MRGPRX2 knockdown LAD2 cells [153]. The anaphylactic effect of sinomenine in vivo was also investigated. The mouse model of PCA showed that sinomenine injection induced extensive paw extravasation and swelling. These inflammatory responses were almost completely absent in mice with MC depletion or MrgprB2 knockdown mice, compared to WT mice [153]. Molecular docking and competitive binding studies showed that sinomenine binds directly to MRGPRX2 [155], most likely at the active site of the receptor (Figure 3) [39,153]. These studies have also determined EC_50_ for sinomenine. For LAD2 cells, EC_50_ was 2.16 µM [32], for MRGPRX2-HEK293 cells it was 1.84 µM and 2.77 ± 0.44 µM ([32] and [153], respectively), and for MrgprB2-HEK293 cells it was 2318 ± 314 µM [153]. The data showed the EC_50_ values to be even lower than those obtained for morphine and MRGPRX2 (4.5–7 µM) [39,156,157].

#### 3.5.2. Piperine

Piperine is another alkaloid with inhibitory properties related to MRGPRX2 [36]. It is found in the fruits of long and black peppers (*Piper longum* and *Piper nigrum*) [152]. Piperine has been reported to suppress both early (degranulation) and late (de novo synthesis of mediators) responses to MC activation [36]. In addition to inhibiting C48/80-induced LAD2 cells degranulation, it also reduced ciprofloxacin and LL-37-induced activation of MCs [36]. Additionally, affinity chromatography methods showed a competitive binding of piperine to MRGPRX2 compared to sinomenine and ciprofloxacin [36,38]. In animal models, piperine ameliorated cutaneous symptoms and systemic anaphylaxis in mice [36]. Piperine could also reduce secretion of IL-31, suggesting that it has alleviating effect on pruritus [36,73]. In addition, the suppressive effect on MC degranulation induced by LL-37 [36], which is abundant in rosacea tissues [8,10], highlights the potential use of piperine in the treatment of this condition. In conclusion, these data indicate that piperine exhibits certain inhibitory properties related to the attenuation of the MC simulation, including drug-induced MC activation leading to allergic reactions. Therefore, further studies are warranted to elucidate its potential as a therapeutic agent in allergic conditions.

**Table 1:** Overview of compounds discussed in this manuscript (further details of the experimental studies are presented in the Supplementary Materials—Table S1).

Compound	Experimental Model or Methods	Primary Outcome Measure	Key Conclusions about Compound Activity	References	MRGPRX2 Inhibition and/or Activation	EC_50_ and/or IC_50_ for MRGPRX2 (Experimental Model and Assay)	C_max_ in Plasma
Salvianolic acid	Molecular docking, molecular dynamics	Inhibition of PI3K and mTOR	A candidate for in vitro experiments in breast cancer studies	[89]	Activation * [37]	EC_50_ = 15.70 ± 4.62 μM (MPMC, β-hexosaminidase release assay) [37]	171.48 ± 9.42 ng/mL ^1^ (0.00024 μM) [158]
Rosmarinic acid	Mouse and rat models	Behavioral tests	Antinociceptive and anti-inflammatory activity	[130]	Inhibition [72] /no effect [35,40] ^2^	IC_50_ = 1.8 mM (MRGPRX2-HEK293 cells, retention time on CMC column) [40] IC_50_ cannot be calculated (MRGPRX2-HEK293 cells, intracellular Ca^2+^ mobilization assay) [35]	
	Carrageenan-induced pleurisy and paw edema tests in rats	Behavioral tests	Potential for anti-inflammatory and antinociceptive activity	[129]			
	PC12 cells	Amyloid β-induced cellular reactive oxygen species generation	A candidate for neuroprotective treatment of Alzheimer’s disease	[159]			162.20 ± 40.20 nmol/L (0.162 mM) [160]
	Mouse model of cardiac fibrosis	Morphological examination, echocardiography	Promising as a therapeutic agent against cardiac fibrosis	[161]			
Baicalin	Mouse model of anxiety/ depression	Depression-like behaviors	Improvement of anxiety/ depression-like behaviors	[162]	Activation * [33,133]	NA	-
	Rat model of peridontitis	Toll-like receptor expression	Potential for treatment of periodontitis	[163]			
	Mouse model	Tumor growth	Potential for treatment of lung cancer	[87]			
Liquiritin	Rat model	Cell viability, inflammatory cytokine expression	Beneficial impact on pressure ulcers	[164]	Inhibition [41]	NA	-
	Rat model	Behavioral tests	Potential for treatment of bone cancer pain	[165]			
	PC12 cells	Expression of proteins involved in signalling pathway	Neuroprotective activity	[166]			
	Diabetic mouse model	α-glucosidase inhibition	Potential for treating diabetes	[167]			
	H9C2 cells	Cell viability level	Cardioprotective effect	[168]			
Fisetin	Male C57bl/6 J mice	Histopathological and serological injury markers	Protection against septic acute kidney injury	[142]	Inhibition [42]	NA	-
	Prostate and lung adenocarcinoma cells	Inhibition of the PI3K/AKT and the mTOR pathways	Potential as adjuvant with chemotherapeutic drugs	[143]			
Osthole	Pulmonary inflammation induced in mice	Inflammatory parameters in BAL fluid	Potential for inhibition of inflammation in chronic obstructive pulmonary disease	[169]	Inhibition [34]/activation [38] ^3^	NA	-
	Mouse model	Itch–scratch response	Antipruritic activity	[170]			
	Mouse monocyte-macrophage cells	Inflammatory mediators’ level	Potential for treatment of ulcerative colitis	[92]			
	Model of middle cerebral artery occlusion in rats	Determination of the infarct area	Potential for neuroprotective therapy in ischemic stroke	[93]			
	Bleomycin induced pulmonary fibrosis in rats	Expression of inflammatory mediators	Beneficial effects in tested model	[171]			
	Cervical cancer cell lines	Cancer cell viability, proliferation, and migration and invasion	Potential as adjuvant treatment for cervical cancer	[172]			
	Human gastric cancer cells	Cell proliferation and apoptosis	Potential for inhibition of gastric cancer cells proliferation	[88]			
	Osteosarcoma cell lines	Cell viability	Potential for osteosarcoma treatment	[173]			
	Tumor-bearing mice	Survival days	Potential for developing antitumor drugs	[174]			
	Diabetic mice	PPAR activation	Potential for treatment of diabetes	[175]			
	Skeletal muscle cells	Expression of AMP-activated protein kinase and glucose transporter 4	Potential for treatment of diabetes	[176]			
Praeruptorin A	Mouse macrophages	Expression of NF-κB-related proteins	Potential as a drug for viral infection	[177]	Activation [38]	NA	-
	Human hepatocellular carcinoma	Migration and invasion of tested cells	Potential as a therapeutic agent in human hepatocellular carcinoma	[178]			
Sinomenine	Rat neuron–glial cultures	Expression of TNF-α, prostaglandin E2, and reactive oxygen species	Potential for treatment of inflammation-mediated neuro-degenerative diseases	[179]	Activation [32,39,43,153,155]	EC_50_ = 2.16 µM (LAD2 cells, intracellular Ca^2+^ mobilization assay) [32] EC_50_ = 1.84 µM (MRGPRX2-HEK293 cells, intracellular Ca^2+^ mobilization assay) [32] EC_50_ = 2.77 ± 0.44 µM (MRGPRX2-HEK293 cells, intracellular Ca^2+^ mobilization assay) [153] EC_50_ = 2318 ± 314 µM (MrgprB2-HEK293 cells, intracellular Ca^2+^ mobilization assay) [153]	
	Rats and mice models	Behavioral tests	Analgesic effect in rodent models	[180]			123 ± 22 ng/mL (0.00037 µM) [181]
	Human bladder cancer cell line	P-glycoprotein expression	A candidate for treatment of bladder cancer	[182]			
	Mouse model of middle cerebral artery occlusion	Brain edema, neuronal apoptosis, neurological deficiency	A candidate for stroke therapy	[183]			
	Microglial cells	Amyloid β-induced levels of reactive oxygen species and nitric oxide	Potential for treatment of Alzheimer’s diseases	[184]			
Piperine	Cervical cancer and non-tumoral cell lines	Cell proliferation, viability, and migration	Potential as complementary treatment in cervical cancer	[185]	Inhibition [36,38]	NA	-

* For these compounds reports of anaphylactoid reactions to injections with them are cited in the main text. ^1^ Maximum concentration in rat plasma. ^2^ Rosmarinic acid has been described in separate studies as inhibitory compound for MRGPRX2 or with no effect on the receptor; for details see Section 3.2.2. ^3^ Osthole has been described as an inhibitory compound for MRGPRX2 and an activator of RBL-2H3 cells with unclear target; for details see Section 3.4.2. Abbreviations: BAL, bronchoalveolar lavage; C_max_, maximum concentration in plasma; EC_50_, half maximal effective concentration; IC_50_, half maximal inhibitory concentration; MPMC, mouse peritoneal mast cells; mTOR, mammalian target of rapamycin; NA, not applicable; NF-κB, nuclear factor kappa-light-chain-enhancer of activated B cells; PI3K, phosphoinositide 3-kinase; PPAR, peroxisome proliferator-activated receptors; TNF, tumor necrosis factor.

## 4. Discussion

In vitro and in vivo studies using animals have demonstrated the involvement of the MRGPRX2 receptor on MCs in numerous physiological and pathological processes—including anaphylactoid responses to various ligands—including FDA-approved drugs, immune responses, host defense against bacteria, tissue homeostasis and repair, nociception and pain, and sleep regulation [11,186]. However, there is a lack of reliable studies in humans. MRGPRX2 is known to be activated by a variety of naturally derived ligands, including phenols, terpenoids, flavonoids, quinones, coumarins, and lignans, as highlighted in the recent literature [9,10]. A wide range of these compounds are used in TCM for the prevention and treatment of various diseases [187]. It should be noted that the estimated number of TCMs is around 12,800 [188]. Despite the increasing use of TCMs worldwide and their therapeutic appeal, its integration into mainstream healthcare continues to be impeded by the absence of strong evidence from an evidence-based medicine (EBM) standpoint. One fundamental limitation is the batch-to-batch variation of the active constituents contained in the herbal formulation used [84].

Nevertheless, TCMs injections are widely used in clinical settings; but ADRs, including the incidence of anaphylaxis, have been increasing annually, posing a serious public health concern [189]. On the other hand, TCM components and other herbal substances have been reported to have an inhibitory effect on MRGPRX2-induced MC stimulation and have been suggested to have protective effects in many skin diseases and pseudoallergic reactions (Figure 1) [9]. Notably, flavonoids, which are typically known for their anti-inflammatory properties (Table 1), exhibit diverse effects on MRGPRX2. Baicalein, for instance, is an exception that can cause pseudoallergic reactions by activating MRGPRX2 [9]; while other flavonoids, which are richer in hydroxyl groups, act as antagonists of this receptor. The most prominent group of the receptor agonists are the opium alkaloids, which include morphine, codeine, sinomenine, and thebaine [4,32,39]. Given that some of these compounds are present in TCMs and have been described to cause anaphylactoid reactions, as have a large number of drugs approved by the FDA, it is important to be aware of the possibility of their occurrence and to manage them appropriately.

On the other hand, some TCM compounds were reported to indicate protective effects against MRGPRX2-dependent anaphylaxis and chronic skin disorders. For instance, one of the candidates could be osthole, a plant-derived coumarin, which has been shown to reduce SP- and LL-37-induced MC degranulation, and to attenuate mouse models of anaphylaxis to SP and LL-37-stimulated rosacea [34]. Similar results were obtained with piperine, which also prevented MCs degranulation to LL-37, but also reduced IL-31 secretion [36], which has been proposed as a key clinical target for the treatment of pruritus [73]. Treatment with fisetin abolished the SP and ovalbumin co-stimulated mouse model of CU [42]. In the mouse model of ACD, rosmarinic acid has been demonstrated to attenuate ACD manifestations and suppress non-histaminergic pruritus by inhibiting MRGPRX2-mediated MC degranulation to CST-14 and by reducing levels of the proinflammatory cytokine IL-13 in mouse tissues [72,74]. In addition, rosmarinic acid and osthole act on the level of MRGPRX2 in the MC, therefore they may have additional suppressing effects in pseudoallergic reactions and skin diseases [34,72].

There are several limitations in the studies presented in this review. All data are based on preclinical studies involving cell lines and mouse models. While animal models with knockdown of MrgprB2, along with in vitro studies using human cell lines and MRGPRX2 knockdown/silencing, have demonstrated the involvement of the aforementioned xenobiotics in MRGPRX2-mediated MC activation/inhibition, conclusive evidence is still needed to confirm whether MRGPRX2 can mediate such effects in humans. While evolutionarily conserved, differences exist between human and mouse MCs. Human MCs demonstrate higher diversity, and the expression of MRGPRX2 can significantly vary among individuals. [23,190] Moreover, there is only approximately 53% overall sequence similarity between mouse and human homologues [191]. Notably, studies have revealed that certain drugs such as ciprofloxacin or levofloxacin activate MRGPRX2 with EC_50_ values 20–35 times lower than its mouse ortholog, MrbprB2. [4] For instance, the studies of sinomenine demonstrated significant difference of EC_50_ in MRGPRX2- and MrgprB2-transfected HEK293 cells (EC_50_ = 2318 µM and EC_50_ = 1.84–2.77 µM, respectively) [32,37]. Moreover, the EC_50_ values can vary between different cell models (cell lines vs. primary cells) [186]. Additionally, it has been observed that several antagonists, including L733060 and aprepitant, inhibit SP-induced activation of mouse Mrgprb2 but do not inhibit human MRGPRX2 [192]. These findings suggest significant species–specific differences between human MRGPRX2 and mouse MrgprB2, indicating that MrgprB2 mutant mice may not be suitable models for screening drugs intended for human use.

In vitro and in vivo animal studies can also not exactly reflect the function of MRGPRX2 in human tissues, because the key role in potential MRGPRX2-mediated anaphylaxis may also depend on the receptor’s biology and the way of drug administration. Due to low affinity of the receptor and thus the relatively high concentration of substance needed to trigger response, the local concentration of the substance may be difficult to achieve. Examples of drugs such as atracurium have been described, whose plasma concentrations after administration are markedly lower than the calculated EC_50_ for MRGPRX2 [186]. MC_TC_, which is found predominantly in the skin, expresses high levels of MRGPRX2. Therefore, the TCMs administration route plays an equally important role. Notably, over 80% of TCM anaphylactoid reactions occur during parenteral administration [189], and might result from high local TCM concentration after injection and subsequent potent stimulation of skin MCs. Another possibility is that the receptor may be activated or inhibited by the same compound, depending on its concentration. This dose-dependent effect is known in the case of some opioid drugs, such as nalbuphine [193], where the agonistic or inhibitory effect depends on the concentration of the drug, as well as the levels and conformation of the receptor [194,195]. Moreover, the absence of specific biomarkers for MRGPRX2 activation in vivo complicates human studies and impedes progress in this field.

The available studies on the interaction of natural products and herbal medicines with MRGPRX2 are considerably limited; therefore, caution is advised when drawing final conclusions.

## 5. Conclusions

Research into exogenous ligands for the MRGPRX2 receptor has grown tremendously in recent years. In addition to some typical groups of drugs, these include numerous substances of natural origin that are used in TCM for therapeutic purposes. In our work we have described representative examples of these. They can show both antagonistic and agonistic effects towards MRGPRX2. However, current data are derived from animal studies and cell lines; and more studies using primary human(ized) models are needed.

## Supplementary Materials

The following supporting information can be downloaded at: https://www.mdpi.com/article/10.3390/jox14010024/s1, Table S1: Methodological details of the studies analysed.

## References

[1] Dispenza M.C., Metcalfe D.D., Olivera A. (2023). Research Advances in Mast Cell Biology and Their Translation into Novel Therapies for Anaphylaxis. J. Allergy Clin. Immunol. Pract..

[2] Jutel M., Agache I., Zemelka-Wiacek M., Akdis M., Chivato T., del Giacco S., Gajdanowicz P., Gracia I.E., Klimek L., Lauerma A. (2023). Nomenclature of allergic diseases and hypersensitivity reactions: Adapted to modern needs: An EAACI position paper. Allergy.

[3] Tatemoto K., Nozaki Y., Tsuda R., Konno S., Tomura K., Furuno M., Ogasawara H., Edamura K., Takagi H., Iwamura H. (2006). Immunoglobulin E-independent activation of mast cell is mediated by Mrg receptors. Biochem. Biophys. Res. Commun..

[4] McNeil B.D., Pundir P., Meeker S., Han L., Undem B.J., Kulka M., Dong X. (2015). Identification of a mast-cell-specific receptor crucial for pseudo-allergic drug reactions. Nature.

[5] Porebski G., Kwiecien K., Pawica M., Kwitniewski M. (2018). Mas-Related G Protein-Coupled Receptor-X2 (MRGPRX2) in Drug Hypersensitivity Reactions. Front. Immunol..

[6] St John A.L., Abraham S.N. (2013). Innate immunity and its regulation by mast cells. J. Immunol..

[7] Ogasawara H., Noguchi M. (2021). Therapeutic Potential of MRGPRX2 Inhibitors on Mast Cells. Cells.

[8] Roy S., Chompunud Na Ayudhya C., Thapaliya M., Deepak V., Ali H. (2021). Multifaceted MRGPRX2: New insight into the role of mast cells in health and disease. J. Allergy Clin. Immunol..

[9] Kumar M., Duraisamy K., Chow B.K. (2021). Unlocking the Non-IgE-Mediated Pseudo-Allergic Reaction Puzzle with Mas-Related G-Protein Coupled Receptor Member X2 (MRGPRX2). Cells.

[10] Baldo B.A. (2023). MRGPRX2, drug pseudoallergies, inflammatory diseases, mechanisms and distinguishing MRGPRX2- and IgE/FcεRI-mediated events. Br. J. Clin. Pharmacol..

[11] Quan P.L., Sabaté-Brescó M., Guo Y., Martín M., Gastaminza G. (2021). The Multifaceted Mas-Related G Protein-Coupled Receptor Member X2 in Allergic Diseases and Beyond. Int. J. Mol. Sci..

[12] West P.W., Bulfone-Paus S. (2022). Mast cell tissue heterogeneity and specificity of immune cell recruitment. Front. Immunol..

[13] Elieh Ali Komi D., Wöhrl S., Bielory L. (2020). Mast Cell Biology at Molecular Level: A Comprehensive Review. Clin. Rev. Allergy Immunol..

[14] Wernersson S., Pejler G. (2014). Mast cell secretory granules: Armed for battle. Nat. Rev. Immunol..

[15] Gordon J.R., Galli S.J. (1991). Release of both preformed and newly synthesized tumor necrosis factor alpha (TNF-alpha)/cachectin by mouse mast cells stimulated via the Fc epsilon RI. A mechanism for the sustained action of mast cell-derived TNF-alpha during IgE-dependent biological responses. J. Exp. Med..

[16] Molderings G.J., Afrin L.B. (2023). A survey of the currently known mast cell mediators with potential relevance for therapy of mast cell-induced symptoms. Naunyn Schmiedebergs Arch. Pharmacol..

[17] Boyce J.A. (2007). Mast cells and eicosanoid mediators: A system of reciprocal paracrine and autocrine regulation. Immunol. Rev..

[18] Vliagoftis H., Befus A.D. (2005). Rapidly changing perspectives about mast cells at mucosal surfaces. Immunol. Rev..

[19] Wasiuk A., de Vries V.C., Hartmann K., Roers A., Noelle R.J. (2009). Mast cells as regulators of adaptive immunity to tumours. Clin. Exp. Immunol..

[20] Bischoff S.C. (2007). Role of mast cells in allergic and non-allergic immune responses: Comparison of human and murine data. Nat. Rev. Immunol..

[21] Oskeritzian C.A., Zhao W., Min H.K., Xia H.Z., Pozez A., Kiev J., Schwartz L.B. (2005). Surface CD88 functionally distinguishes the MCTC from the MCT type of human lung mast cell. J. Allergy Clin. Immunol..

[22] Krishnaswamy G., Ajitawi O., Chi D.S. (2006). The human mast cell: An overview. Methods Mol. Biol..

[23] Tauber M., Basso L., Martin J., Bostan L., Pinto M.M., Thierry G.R., Houmadi R., Serhan N., Loste A., Blériot C. (2023). Landscape of mast cell populations across organs in mice and humans. J. Exp. Med..

[24] Fujisawa D., Kashiwakura J., Kita H., Kikukawa Y., Fujitani Y., Sasaki-Sakamoto T., Kuroda K., Nunomura S., Hayama K., Terui T. (2014). Expression of Mas-related gene X2 on mast cells is upregulated in the skin of patients with severe chronic urticaria. J. Allergy Clin. Immunol..

[25] Pyatilova P., Ashry T., Luo Y., He J., Bonnekoh H., Jiao Q., Moñino-Romero S., Hu M., Scheffel J., Frischbutter S. (2022). The Number of MRGPRX2-Expressing Cells Is Increased in Skin Lesions of Patients with Indolent Systemic Mastocytosis, but Is Not Linked to Symptom Severity. Front. Immunol..

[26] Manorak W., Idahosa C., Gupta K., Roy S., Panettieri R., Ali H. (2018). Upregulation of Mas-related G Protein coupled receptor X2 in asthmatic lung mast cells and its activation by the novel neuropeptide hemokinin-1. Respir. Res..

[27] Ray P., Torck A., Quigley L., Wangzhou A., Neiman M., Rao C., Lam T., Kim J.Y., Kim T.H., Zhang M.Q. (2018). Comparative transcriptome profiling of the human and mouse dorsal root ganglia: An RNA-seq-based resource for pain and sensory neuroscience research. Pain.

[28] Kühn H., Kolkhir P., Babina M., Düll M., Frischbutter S., Fok J.S., Jiao Q., Metz M., Scheffel J., Wolf K. (2021). Mas-related G protein-coupled receptor X2 and its activators in dermatologic allergies. J. Allergy Clin. Immunol..

[29] Arifuzzaman M., Mobley Y.R., Choi H.W., Bist P., Salinas C.A., Brown Z.D., Chen S.L., Staats H.F., Abraham S.N. (2019). MRGPR-mediated activation of local mast cells clears cutaneous bacterial infection and protects against reinfection. Sci. Adv..

[30] Seldeslachts A., Peigneur S., Mebs D., Tytgat J. (2022). Unraveling the venom chemistry with evidence for histamine as key regulator in the envenomation by caterpillar *Automeris zaruma*. Front. Immunol..

[31] Wang L., Hu G.Z., Lu Y., Jiang S.J., Qi J., Su H. (2022). Anti-pseudo-allergic components in licorice extract inhibit mast cell degranulation and calcium influx. Chin. J. Nat. Med..

[32] Lei P., Liu Y., Ding Y., Su X., Liang J., Chen H., Ma W. (2022). Thebaine induces anaphylactic reactions via the MRGPRX2 receptor pathway on mast cells. Cell. Immunol..

[33] Wang J., Zhang Y., Che D., Zeng Y., Wu Y., Qin Q., Wang N. (2020). Baicalin induces Mrgprb2-dependent pseudo-allergy in mice. Immunol. Lett..

[34] Callahan B.N., Kammala A.K., Syed M., Yang C., Occhiuto C.J., Nellutla R., Chumanevich A.P., Oskeritzian C.A., Das R., Subramanian H. (2020). Osthole, a Natural Plant Derivative Inhibits MRGPRX2 Induced Mast Cell Responses. Front. Immunol..

[35] Yang L., Zeng Y., Wang J., Zhang Y., Hou Y., Qin Q., Ma W., Wang N. (2020). Discovery and analysis the anti-pseudo-allergic components from *Perilla frutescens* leaves by overexpressed MRGPRX2 cell membrane chromatography coupled with HPLC-ESI-IT-TOF system. J. Pharm. Pharmacol..

[36] Qiao C., Hu S., Che D., Wang J., Gao J., Ma R., Jiang W., Zhang T., Liu R. (2020). The anti-anaphylactoid effects of Piperine through regulating MAS-related G protein-coupled receptor X2 activation. Phytother. Res..

[37] Lin Y., Wang C., Hou Y., Sun W., Che D., Yang L., Zhang T., Sun M., He H., He L. (2018). Simultaneous identification of three pseudoallergic components in Danshen injection by using high-expression Mas-related G protein coupled receptor X2 cell membrane chromatography coupled online to HPLC-ESI-MS/MS. J. Sep. Sci..

[38] Han S., Lv Y., Kong L., Sun Y., Fu J., Li L., He L. (2018). Simultaneous identification of the anaphylactoid components from traditional Chinese medicine injections using rat basophilic leukemia 2H3 and laboratory of allergic disease 2 dual-mixed/cell membrane chromatography model. Electrophoresis.

[39] Lansu K., Karpiak J., Liu J., Huang X.P., McCorvy J.D., Kroeze W.K., Che T., Nagase H., Carroll F.I., Jin J. (2017). In silico design of novel probes for the atypical opioid receptor MRGPRX2. Nat. Chem. Biol..

[40] Adhikari N., Shim W.S. (2022). Caffeic acid phenethyl ester inhibits pseudo-allergic reactions via inhibition of MRGPRX2/MrgprB2-dependent mast cell degranulation. Arch. Pharm. Res..

[41] Wang L., Huang C., Li Z., Hu G., Qi J., Fan Z. (2023). Liquiritin inhibits MRGPRX2-mediated pseudo-allergy through the PI3K/AKT and PLCγ signaling pathways. Heliyon.

[42] Zhang Y., Huang Y., Dang B., Hu S., Zhao C., Wang Y., Yuan Y., Liu R. (2023). Fisetin alleviates chronic urticaria by inhibiting mast cell activation via MRGPRX2. J. Pharm. Pharmacol..

[43] Jia Q., Fu J., Gao C., Wang H., Wang S., Liang P., Han S., Lv Y., He L. (2022). MrgX2-SNAP-tag/cell membrane chromatography model coupled with liquid chromatography-mass spectrometry for anti-pseudo-allergic compound screening in Arnebiae Radix. Anal. Bioanal. Chem..

[44] Liu R., Hu S., Ding Y., Wang J., Wang Y., Gao J., He L. (2021). Dictamnine is an effective anti-anaphylactoid compound acting via the MrgX2 receptor located on mast cells. Phytother. Res..

[45] Sun W., Wang S., Liang P., Zhou H., Zhang L., Jia Q., Fu J., Lv Y., Han S. (2021). Pseudo-allergic compounds screened from Shengmai injection by using high-expression Mas-related G protein-coupled receptor X2 cell membrane chromatography online coupled with liquid chromatography and mass spectrometry. J. Sep. Sci..

[46] Wang N., Wang J., Zhang Y., Zeng Y., Hu S., Bai H., Hou Y., Wang C., He H., He L. (2021). Imperatorin ameliorates mast cell-mediated allergic airway inflammation by inhibiting MRGPRX2 and CamKII/ERK signaling pathway. Biochem. Pharmacol..

[47] Lin Y., Xu J., Jia Q., Sun W., Fu J., Lv Y., Han S. (2020). Cell membrane chromatography coupled online with LC-MS to screen anti-anaphylactoid components from *Magnolia biondii* Pamp. targeting on Mas-related G protein-coupled receptor X2. J. Sep. Sci..

[48] Wang J., Zhang Y., Wang J., Liu R., Zhang G., Dong K., Zhang T. (2020). Paeoniflorin inhibits MRGPRX2-mediated pseudo-allergic reaction via calcium signaling pathway. Phytother. Res..

[49] Jia Q., Sun W., Zhang L., Fu J., Lv Y., Lin Y., Han S. (2019). Screening the anti-allergic components in Saposhnikoviae Radix using high-expression Mas-related G protein-coupled receptor X2 cell membrane chromatography online coupled with liquid chromatography and mass spectrometry. J. Sep. Sci..

[50] Ding Y., Che D., Li C., Cao J., Wang J., Ma P., Zhao T., An H., Zhang T. (2019). Quercetin inhibits Mrgprx2-induced pseudo-allergic reaction via PLCγ-IP3R related Ca^2+^ fluctuations. Int. Immunopharmacol..

[51] Lin Y., Lv Y., Fu J., Jia Q., Han S. (2018). A high expression Mas-related G protein coupled receptor X2 cell membrane chromatography coupled with liquid chromatography and mass spectrometry method for screening potential anaphylactoid components in kudiezi injection. J. Pharm. Biomed. Anal..

[52] Hou Y., Che D., Ma P., Zhao T., Zeng Y., Wang N. (2018). Anti-pseudo-allergy effect of isoliquiritigenin is MRGPRX2-dependent. Immunol. Lett..

[53] Wang N., Che D., Zhang T., Liu R., Cao J., Wang J., Zhao T., Ma P., Dong X., He L. (2018). Saikosaponin A inhibits compound 48/80-induced pseudo-allergy via the Mrgprx2 pathway in vitro and in vivo. Biochem. Pharmacol..

[54] Johnson T., Siegel D. (2014). Complanadine A, a selective agonist for the Mas-related G protein-coupled receptor X2. Bioorg. Med. Chem. Lett..

[55] Yao C., Ye W., Chen M. (2023). Inhibition of Mast Cell Degranulation in Atopic Dermatitis by Celastrol through Suppressing MRGPRX2. Dis. Markers.

[56] Ye F., Jiang Y., Zhang J., Zong Y., Yu M., Chen C., Zhu C., Yang Y., Jia K., Chen G. (2022). Water Extract of *Senecio scandens* Buch.Ham Ameliorates Pruritus by Inhibiting MrgprB2 Receptor. J. Inflamm. Res..

[57] Jiang Y., Zong Y., Du Y., Zhang M., Ye F., Zhang J., Yang Y., Zhu C., Tang Z. (2023). Curcumin inhibits the pruritus in mice through mast cell MrgprB2 receptor. Inflamm. Res..

[58] Cao J., Wang Y., Hu S., Ding Y., Jia Q., Zhu J., An H. (2020). Kaempferol ameliorates secretagogue-induced pseudo-allergic reactions via inhibiting intracellular calcium fluctuation. J. Pharm. Pharmacol..

[59] Liu R., Zhao T., Che D., Cao J., Wang J., Lv Y., Ma P., Ding Y., Wang N., Wang X. (2018). The anti-anaphylactoid effects of hydroxysafflor yellow A on the suppression of mast cell Ca^2+^ influx and degranulation. Phytomedicine.

[60] Cao C., Roth B.L. (2023). The structure, function, and pharmacology of MRGPRs. Trends Pharmacol. Sci..

[61] Gupta K., Subramanian H., Klos A., Ali H. (2012). Phosphorylation of C3a receptor at multiple sites mediates desensitization, β-arrestin-2 recruitment and inhibition of NF-κB activity in mast cells. PLoS ONE.

[62] Cahill T.J., Thomsen A.R., Tarrasch J.T., Plouffe B., Nguyen A.H., Yang F., Huang L.Y., Kahsai A.W., Bassoni D.L., Gavino B.J. (2017). Distinct conformations of GPCR-β-arrestin complexes mediate desensitization, signaling, and endocytosis. Proc. Natl. Acad. Sci. USA.

[63] Mi Y.N., Ping N.N., Cao Y.X. (2021). Ligands and Signaling of Mas-Related G Protein-Coupled Receptor-X2 in Mast Cell Activation. Rev. Physiol. Biochem. Pharmacol..

[64] Chompunud Na Ayudhya C., Roy S., Alkanfari I., Ganguly A., Ali H. (2019). Identification of Gain and Loss of Function Missense Variants in MRGPRX2’s Transmembrane and Intracellular Domains for Mast Cell Activation by Substance P. Int. J. Mol. Sci..

[65] Venkatakrishnan A.J., Deupi X., Lebon G., Heydenreich F.M., Flock T., Miljus T., Balaji S., Bouvier M., Veprintsev D.B., Tate C.G. (2016). Diverse activation pathways in class A GPCRs converge near the G-protein-coupling region. Nature.

[66] Chompunud Na Ayudhya C., Amponnawarat A., Ali H. (2021). Substance P Serves as a Balanced Agonist for MRGPRX2 and a Single Tyrosine Residue Is Required for β-Arrestin Recruitment and Receptor Internalization. Int. J. Mol. Sci..

[67] Murakami T., Suzuki K., Niyonsaba F., Tada H., Reich J., Tamura H., Nagaoka I. (2018). MrgX2-mediated internalization of LL-37 and degranulation of human LAD2 mast cells. Mol. Med. Rep..

[68] Song J., Xian D., Yang L., Xiong X., Lai R., Zhong J. (2018). Pruritus: Progress toward Pathogenesis and Treatment. Biomed. Res. Int..

[69] Cao C., Kang H.J., Singh I., Chen H., Zhang C., Ye W., Hayes B.W., Liu J., Gumpper R.H., Bender B.J. (2021). Structure, function and pharmacology of human itch GPCRs. Nature.

[70] Meixiong J., Anderson M., Limjunyawong N., Sabbagh M.F., Hu E., Mack M.R., Oetjen L.K., Wang F., Kim B.S., Dong X. (2019). Activation of Mast-Cell-Expressed Mas-Related G-Protein-Coupled Receptors Drives Non-histaminergic Itch. Immunity.

[71] Kolkhir P., Pyatilova P., Ashry T., Jiao Q., Abad-Perez A.T., Altrichter S., Vera Ayala C.E., Church M.K., He J., Lohse K. (2022). Mast cells, cortistatin, and its receptor, MRGPRX2, are linked to the pathogenesis of chronic prurigo. J. Allergy Clin. Immunol..

[72] Ding Y., Ma T., Zhang Y., Zhao C., Wang C., Wang Z. (2023). Rosmarinic acid ameliorates skin inflammation and pruritus in allergic contact dermatitis by inhibiting mast cell-mediated MRGPRX2/PLCγ1 signaling pathway. Int. Immunopharmacol..

[73] Kabashima K., Irie H. (2021). Interleukin-31 as a Clinical Target for Pruritus Treatment. Front. Med..

[74] Zheng T., Oh M.H., Oh S.Y., Schroeder J.T., Glick A.B., Zhu Z. (2009). Transgenic expression of interleukin-13 in the skin induces a pruritic dermatitis and skin remodeling. J. Investig. Dermatol..

[75] Facheris P., Jeffery J., Del Duca E., Guttman-Yassky E. (2023). The translational revolution in atopic dermatitis: The paradigm shift from pathogenesis to treatment. Cell. Mol. Immunol..

[76] Nassau S., Fonacier L. (2020). Allergic Contact Dermatitis. Med. Clin. N. Am..

[77] Zuberbier T., Abdul Latiff A.H., Abuzakouk M., Aquilina S., Asero R., Baker D., Ballmer-Weber B., Bangert C., Ben-Shoshan M., Bernstein J.A. (2022). The international EAACI/GA^2^LEN/EuroGuiDerm/APAAACI guideline for the definition, classification, diagnosis, and management of urticaria. Allergy.

[78] Greiwe J., Bernstein J.A. (2017). Therapy of antihistamine-resistant chronic spontaneous urticaria. Expert. Rev. Clin. Immunol..

[79] Li L., Liu R., Peng C., Chen X., Li J. (2022). Pharmacogenomics for the efficacy and side effects of antihistamines. Exp. Dermatol..

[80] Karalliedde L.D., Kappagoda C.T. (2009). The challenge of traditional Chinese medicines for allopathic practitioners. Am. J. Physiol. Heart Circ. Physiol..

[81] Chan H.H.L., Ng T. (2020). Traditional Chinese Medicine (TCM) and Allergic Diseases. Curr. Allergy Asthma Rep..

[82] Yang Y., Li X., Chen G., Xian Y., Zhang H., Wu Y., Yang Y., Wu J., Wang C., He S. (2023). Traditional Chinese medicine compound (Tongxinluo) and clinical outcomes of patients with acute myocardial infarction: The CTS-AMI randomized clinical trial. JAMA.

[83] Sackett D.L., Rosenberg W.M.C., Gray J.A.M., Haynes R.B., Richardson W.S. (1996). Evidence based medicine: What it is and what it isn’t. Br. Med. J..

[84] Fung F.Y., Linn Y.C. (2015). Developing traditional chinese medicine in the era of evidence-based medicine: Current evidences and challenges. Evid.-Based Complement. Alternat. Med..

[85] Ng A.W.T., Poon S.L., Huang M.N., Lim J.Q., Boot A., Yu W., Suzuki Y., Thangaraju S., Ng C.C.Y., Tan P. (2017). Aristolochic acids and their derivatives are widely implicated in liver cancers in Taiwan and throughout Asia. Sci. Transl. Med..

[86] Li J., Liu Z., Chen R., Hu D., Li W., Li X., Chen X., Huang B. (2014). The quality of reports of randomized clinical trials on traditional Chinese medicine treatments: A systematic review of articles indexed in the China National Knowledge Infrastructure database from 2005 to 2012. BMC Complement. Altern. Med..

[87] Cathcart M.C., Useckaite Z., Drakeford C., Semik V., Lysaght J., Gately K., O’Byrne K.J., Pidgeon G.P. (2016). Anti-cancer effects of baicalein in non-small cell lung cancer in-vitro and in-vivo. BMC Cancer.

[88] Xu X., Liu X., Zhang Y. (2018). Osthole inhibits gastric cancer cell proliferation through regulation of PI3K/AKT. PLoS ONE.

[89] Kumar B.H., Manandhar S., Choudhary S.S., Priya K., Gujaran T.V., Mehta C.H., Nayak U.Y., Pai K.S.R. (2023). Identification of phytochemical as a dual inhibitor of PI3K and mTOR: A structure-based computational approach. Mol. Divers..

[90] Orange Book: Approved Drug Products with Therapeutic Equivalence Evaluations. https://www.accessdata.fda.gov/scripts/cder/ob/index.cfm.

[91] European Medicined Agency. https://www.ema.europa.eu/en/medicines.

[92] Fan H., Gao Z., Ji K., Li X., Wu J., Liu Y., Wang X., Liang H., Liu Y., Li X. (2019). The in vitro and in vivo anti-inflammatory effect of osthole, the major natural coumarin from *Cnidium monnieri* (L.) Cuss, via the blocking of the activation of the NF-κB and MAPK/p38 pathways. Phytomedicine.

[93] Li F., Gong Q., Wang L., Shi J. (2012). Osthole attenuates focal inflammatory reaction following permanent middle cerebral artery occlusion in rats. Biol. Pharm. Bull..

[94] Wang J., Fu Y., Wei Z., He X., Shi M., Kou J., Zhou E., Liu W., Yang Z., Guo C. (2017). Anti-asthmatic activity of osthole in an ovalbumin-induced asthma murine model. Respir. Physiol. Neurobiol..

[95] Chiang C.Y., Lee C.C., Fan C.K., Huang H.M., Chiang B.L., Lee Y.L. (2017). Osthole treatment ameliorates Th2-mediated allergic asthma and exerts immunomodulatory effects on dendritic cell maturation and function. Cell. Mol. Immunol..

[96] Matsuda H., Tomohiro N., Ido Y., Kubo M. (2002). Anti-allergic effects of cnidii monnieri fructus (dried fruits of *Cnidium monnieri*) and its major component, osthol. Biol. Pharm. Bull..

[97] Fu X., Hong C. (2019). Osthole attenuates mouse atopic dermatitis by inhibiting thymic stromal lymphopoietin production from keratinocytes. Exp. Dermatol..

[98] Dias M.C., Pinto D.C.G.A., Silva A.M.S. (2021). Plant Flavonoids: Chemical Characteristics and Biological Activity. Molecules.

[99] Hada Y., Uchida H.A., Wada J. (2021). Fisetin Attenuates Lipopolysaccharide-Induced Inflammatory Responses in Macrophage. Biomed. Res. Int..

[100] Jo W.R., Park H.J. (2017). Antiallergic effect of fisetin on IgE-mediated mast cell activation in vitro and on passive cutaneous anaphylaxis (PCA). J. Nutr. Biochem..

[101] Huajuan J., Xulong H., Bin X., Yue W., Yongfeng Z., Chaoxiang R., Jin P. (2023). Chinese herbal injection for cardio-cerebrovascular disease: Overview and challenges. Front. Pharmacol..

[102] Shao H., Li M., Chen F., Chen L., Jiang Z., Zhao L. (2018). The Efficacy of Danshen Injection as Adjunctive Therapy in Treating Angina Pectoris: A Systematic Review and Meta-Analysis. Heart Lung Circ..

[103] Xu M., Liu L., Qi C., Deng B., Cai X. (2008). Sinomenine versus NSAIDs for the treatment of rheumatoid arthritis: A systematic review and meta-analysis. Planta Med..

[104] Bajek-Bil A., Chmiel M., Włoch A., Stompor-Gorący M. (2023). Baicalin-Current Trends in Detection Methods and Health-Promoting Properties. Pharmaceuticals.

[105] Wahab S., Annadurai S., Abullais S.S., Das G., Ahmad W., Ahmad M.F., Kandasamy G., Vasudevan R., Ali M.S., Amir M. (2021). *Glycyrrhiza glabra* (Licorice): A Comprehensive Review on Its Phytochemistry, Biological Activities, Clinical Evidence and Toxicology. Plants.

[106] Sarkhail P., Shafiee A., Sarkheil P. (2013). Biological activities and pharmacokinetics of praeruptorins from *Peucedanum* species: A systematic review. Biomed Res. Int..

[107] Meghwal M., Goswami T.K. (2013). Piper nigrum and piperine: An update. Phytother. Res..

[108] Yu H., Qiu J.F., Ma L.J., Hu Y.J., Li P., Wan J.B. (2017). Phytochemical and phytopharmacological review of *Perilla frutescens* L. (Labiatae), a traditional edible-medicinal herb in China. Food Chem. Toxicol..

[109] Wollam J., Solomon M., Villescaz C., Anderson S., Freeman D., Vasquez A., Pisacane C., Vest A., Napora J., Charlot B. (2023). MRGPRX2 small molecule antagonists potently inhibit agonist-induced skin mast cell degranulation. Allergy.

[110] ClinicalTrials.gov Nationial Library of Medicine. MEDLINE. https://clinicaltrials.gov.

[111] Rana A., Samtiya M., Dhewa T., Mishra V., Aluko R.E. (2022). Health benefits of polyphenols: A concise review. J. Food Biochem..

[112] Vaisman N., Niv E. (2015). Daily consumption of red grape cell powder in a dietary dose improves cardiovascular parameters: A double blind, placebo-controlled, randomized study. Int. J. Food Sci. Nutr..

[113] Ogawa S., Matsumae T., Kataoka T., Yazaki Y., Yamaguchi H. (2013). Effect of acacia polyphenol on glucose homeostasis in subjects with impaired glucose tolerance: A randomized multicenter feeding trial. Exp. Ther. Med..

[114] Moorthy M., Chaiyakunapruk N., Jacob S.A., Palanisamy U.D. (2020). Prebiotic potential of polyphenols, its effect on gut microbiota and anthropometric/clinical markers: A systematic review of randomised controlled trials. Trends Food Sci. Technol..

[115] Magyar K., Halmosi R., Palfi A., Feher G., Czopf L., Fulop A., Battyany I., Sumegi B., Toth K., Szabados E. (2012). Cardioprotection by resveratrol: A human clinical trial in patients with stable coronary artery disease. Clin. Hemorheol. Microcirc..

[116] Henning S.M., Wang P., Said J.W., Huang M., Grogan T., Elashoff D., Carpenter C.L., Heber D., Aronson W.J. (2015). Randomized clinical trial of brewed green and black tea in men with prostate cancer prior to prostatectomy. Prostate.

[117] González Arbeláez L.F., Ciocci Pardo A., Fantinelli J.C., Schinella G.R., Mosca S.M., Ríos J.L. (2018). Cardioprotection and natural polyphenols: An update of clinical and experimental studies. Food Funct..

[118] Chiva-Blanch G., Urpi-Sarda M., Ros E., Valderas-Martinez P., Casas R., Arranz S., Guillén M., Lamuela-Raventós R.M., Llorach R., Andres-Lacueva C. (2013). Effects of red wine polyphenols and alcohol on glucose metabolism and the lipid profile: A randomized clinical trial. Clin. Nutr..

[119] Cao H., Ou J., Chen L., Zhang Y., Szkudelski T., Delmas D., Daglia M., Xiao J. (2019). Dietary polyphenols and type 2 diabetes: Human Study and Clinical Trial. Crit. Rev. Food Sci. Nutr..

[120] Lin Y., Wang J., Hou Y., Fu J., Wei D., Jia Q., Lv Y., Wang C., Han S., He L. (2019). Isosalvianolic acid C-induced pseudo-allergic reactions via the mast cell specific receptor MRGPRX2. Int. Immunopharmacol..

[121] Zhu C., Cao H., Zhou X., Dong C., Luo J., Zhang C., Liu J., Ling Y. (2013). Meta-analysis of the clinical value of Danshen injection and Huangqi injection in liver cirrhosis. Evid.-Based Complement. Alternat. Med..

[122] Guo S., Wu J., Ni M., Jia S., Zhang J., Zhou W., Liu X., Wang M., Zhang X. (2020). Comparative Efficacy of Danshen Class Injections for Treating Acute Coronary Syndrome: A Multidimensional Bayesian Network Meta-Analysis of Randomized Controlled Trials. Front. Pharmacol..

[123] Wang L., Yuan Q., Marshall G., Cui X., Cheng L., Li Y., Shang H., Zhang B., Li Y. (2010). Adverse drug reactions and adverse events of 33 varieties of traditional Chinese medicine injections on National Essential medicines List (2004 edition) of China: An overview on published literatures. J. Evid.-Based Med..

[124] Guo Y.J., Wang D.W., Meng L., Wang Y.Q. (2015). Analysis of anaphylactic shock caused by 17 types of traditional Chinese medicine injections used to treat cardiovascular and cerebrovascular diseases. Biomed. Res. Int..

[125] Xu J., Zeng S., Chen X., Qu H. (2011). Isolation and identification of degradation products of salvianolic acid A by NMR and LC-MS. Fitoterapia.

[126] Mahalakshmi B., Huang C.Y., Lee S.D., Maurya N., Kiefer R., Kumar B.V. (2021). Review of Danshen: From its metabolism to possible mechanisms of its biological activities. J. Funct. Foods.

[127] Zhang L., Chen H.-J., Wang X.-W. (2008). One case of *Salvia miltiorrhiza* injection intravenous drip too fast induced allergic reaction. Nurs. Pract. Res..

[128] Jiang H.-Q., Chen S.-Y. (2008). One case of high concentration Salvia miltiorrhiza injection induced hypovolemic shock. Strait Pharm. J..

[129] Takaki I., Bersani-Amado L.E., Vendruscolo A., Sartoretto S.M., Diniz S.P., Bersani-Amado C.A., Cuman R.K. (2008). Anti-inflammatory and antinociceptive effects of *Rosmarinus officinalis* L. essential oil in experimental animal models. J. Med. Food.

[130] González-Trujano M.E., Peña E.I., Martínez A.L., Moreno J., Guevara-Fefer P., Déciga-Campos M., López-Muñoz F.J. (2007). Evaluation of the antinociceptive effect of *Rosmarinus officinalis* L. using three different experimental models in rodents. J. Ethnopharmacol..

[131] Chen W.P., Jin G.J., Xiong Y., Hu P.F., Bao J.P., Wu L.D. (2018). Rosmarinic acid down-regulates NO and PGE2 expression via MAPK pathway in rat chondrocytes. J. Cell. Mol. Med..

[132] Ding Y., Dang B., Zhang Y., Hu S., Wang Y., Zhao C., Zhang T., Gao Z. (2023). Paeonol attenuates Substance P-induced urticaria by inhibiting Src kinase phosphorylation in mast cells. Cell. Immunol..

[133] Han S., Lv Y., Kong L., Che D., Liu R., Fu J., Cao J., Wang J., Wang C., He H. (2017). Use of the relative release index for histamine in LAD2 cells to evaluate the potential anaphylactoid effects of drugs. Sci. Rep..

[134] Maleki S.J., Crespo J.F., Cabanillas B. (2019). Anti-inflammatory effects of flavonoids. Food Chem..

[135] Xue Z., Zhang Y., Zeng Y., Hu S., Bai H., Wang J., Jing H., Wang N. (2021). Licochalcone A inhibits MAS-related GPR family member X2-induced pseudo-allergic reaction by suppressing nuclear migration of nuclear factor-κB. Phytother. Res..

[136] Zhang Q., Cai C., Wang P., Ding N., Gao J., Zhang Y., Ma Z., Lei H., Li Q., Jian L. (2017). Baicalin and rutin are major constituents in Shuanghuanglian injection involving anaphylactoid reaction. J. Tradit. Chin. Med..

[137] Tan L., Li M., Lin Y. (2019). Safety Concerns of Traditional Chinese Medicine Injections Used in Chinese Children. Evid.-Based Complement. Alternat. Med..

[138] Gao Y., Hou R., Han Y., Fei Q., Cai R., Qi Y. (2018). Shuang-Huang-Lian injection induces an immediate hypersensitivity reaction via C5a but not IgE. Sci. Rep..

[139] Cheng M., Zhang J., Yang L., Shen S., Li P., Yao S., Qu H., Li J., Yao C., Wei W. (2021). Recent advances in chemical analysis of licorice (Gan-Cao). Fitoterapia.

[140] Kashyap D., Sharma A., Sak K., Tuli H.S., Buttar H.S., Bishayee A. (2018). Fisetin: A bioactive phytochemical with potential for cancer prevention and pharmacotherapy. Life Sci..

[141] Terahara N. (2015). Flavonoids in foods: A review. Nat. Prod. Commun..

[142] Ren Q., Guo F., Tao S., Huang R., Ma L., Fu P. (2020). Flavonoid fisetin alleviates kidney inflammation and apoptosis via inhibiting Src-mediated NF-κB p65 and MAPK signaling pathways in septic AKI mice. Biomed. Pharmacother..

[143] Adhami V.M., Syed D.N., Khan N., Mukhtar H. (2012). Dietary flavonoid fisetin: A novel dual inhibitor of PI3K/Akt and mTOR for prostate cancer management. Biochem. Pharmacol..

[144] Chamcheu J.C., Esnault S., Adhami V.M., Noll A.L., Banang-Mbeumi S., Roy T., Singh S.S., Huang S., Kousoulas K.G., Mukhtar H. (2019). Fisetin, a 3,7,3’,4’-Tetrahydroxyflavone Inhibits the PI3K/Akt/mTOR and MAPK Pathways and Ameliorates Psoriasis Pathology in 2D and 3D Organotypic Human Inflammatory Skin Models. Cells.

[145] Sharifi-Rad J., Cruz-Martins N., López-Jornet P., Lopez E.P., Harun N., Yeskaliyeva B., Beyatli A., Sytar O., Shaheen S., Sharopov F. (2021). Natural Coumarins: Exploring the Pharmacological Complexity and Underlying Molecular Mechanisms. Oxid. Med. Cell. Longev..

[146] Torres R., Faini F., Modak B., Urbina F., Labbé C., Guerrero J. (2006). Antioxidant activity of coumarins and flavonols from the resinous exudate of *Haplopappus multifolius*. Phytochemistry.

[147] Kirsch G., Abdelwahab A.B., Chaimbault P. (2016). Natural and Synthetic Coumarins with Effects on Inflammation. Molecules.

[148] Al-Amiery A.A., Kadhum A.A., Mohamad A.B. (2012). Antifungal activities of new coumarins. Molecules.

[149] Warhi T., Sabt A., Elkaeed E.B., Eldehna W.M. (2020). Recent advancements of coumarin-based anticancer agents: An up-to-date review. Bioorg. Chem..

[150] Bubols G.B., Vianna D.D.R., Medina-Remon A., von Poser G., Maria Lamuela-Raventos R., Lucia Eifler-Lima V., Cristina Garcia S. (2013). The antioxidant activity of coumarins and flavonoids. Mini Rev. Med. Chem..

[151] Sarker S.D., Nahar L. (2017). Progress in the Chemistry of Naturally Occurring Coumarins. Prog. Chem. Org. Nat. Prod..

[152] Bhambhani S., Kondhare K.R., Giri A.P. (2021). Diversity in Chemical Structures and Biological Properties of Plant Alkaloids. Molecules.

[153] Liu R., Che D., Zhao T., Pundir P., Cao J., Lv Y., Wang J., Ma P., Fu J., Wang N. (2017). MRGPRX2 is essential for sinomenine hydrochloride induced anaphylactoid reactions. Biochem. Pharmacol..

[154] Chen D.P., Wong C.K., Leung P.C., Fung K.P., Lau C.B., Lau C.P., Li E.K., Tam L.S., Lam C.W. (2011). Anti-inflammatory activities of Chinese herbal medicine sinomenine and Liang Miao San on tumor necrosis factor-α-activated human fibroblast-like synoviocytes in rheumatoid arthritis. J. Ethnopharmacol..

[155] Wei D., Hu T., Hou Y.J., Wang X.J., Lu J.Y., Ge S., Wang C., He H.Z. (2021). MRGPRX2 is critical for clozapine induced pseudo-allergic reactions. Immunopharmacol. Immunotoxicol..

[156] Zhang T., Liu R., Che D., Pundir P., Wang N., Han S., Cao J., Lv Y., Dong H., Fang F. (2019). A Mast Cell–Specific Receptor Is Critical for Granuloma Induced by Intrathecal Morphine Infusion. J. Immunol..

[157] Akuzawa N., Obinata H., Izumi T., Takeda S. (2009). Morphine Is an Exogenous Ligand for MrgX2, a G Protein-Coupled Receptor for Cortistatin. J. Cell Anim. Biol..

[158] Song J., Zhang W., Sun J., Zhang X., Xu X., Zhang L., Feng Z., Du G. (2016). Determination of salvianolic acid C in rat plasma using liquid chromatography-mass spectrometry and its application to pharmacokinetic study. Biomed. Chromatogr..

[159] Rong H., Liang Y., Niu Y. (2018). Rosmarinic acid attenuates β-amyloid-induced oxidative stress via Akt/GSK-3β/Fyn-mediated Nrf2 activation in PC12 cells. Free Radic. Biol. Med..

[160] Hitl M., Kladar N., Gavarić N., Božin B. (2021). Rosmarinic Acid-Human Pharmacokinetics and Health Benefits. Planta Med..

[161] Zhang X., Ma Z.G., Yuan Y.P., Xu S.C., Wei W.Y., Song P., Kong C.Y., Deng W., Tang Q.Z. (2018). Rosmarinic acid attenuates cardiac fibrosis following long-term pressure overload via AMPKα/Smad3 signaling. Cell Death Dis..

[162] Zhang K., Pan X., Wang F., Ma J., Su G., Dong Y., Yang J., Wu C. (2016). Baicalin promotes hippocampal neurogenesis via SGK1- and FKBP5-mediated glucocorticoid receptor phosphorylation in a neuroendocrine mouse model of anxiety/depression. Sci. Rep..

[163] Sun J.Y., Li D.L., Dong Y., Zhu C.H., Liu J., Li J.D., Zhou T., Gou J.Z., Li A., Zang W.J. (2016). Baicalin inhibits toll-like receptor 2/4 expression and downstream signaling in rat experimental periodontitis. Int. Immunopharmacol..

[164] Yang X., Dang X., Zhang X., Zhao S. (2021). Liquiritin reduces lipopolysaccharide-aroused HaCaT cell inflammation damage via regulation of microRNA-31/MyD88. Int. Immunopharmacol..

[165] Ni H., Xu M., Xie K., Fei Y., Deng H., He Q., Wang T., Liu S., Zhu J., Xu L. (2020). Liquiritin Alleviates Pain Through Inhibiting CXCL1/CXCR2 Signaling Pathway in Bone Cancer Pain Rat. Front. Pharmacol..

[166] Li X., Qin X., Tian J., Gao X., Wu X., Du G., Zhou Y. (2020). Liquiritin protects PC12 cells from corticosterone-induced neurotoxicity via regulation of metabolic disorders, attenuation ERK1/2-NF-κB pathway, activation Nrf2-Keap1 pathway, and inhibition mitochondrial apoptosis pathway. Food Chem. Toxicol..

[167] Zhang W., Li T., Zhang X.J., Zhu Z.Y. (2020). Hypoglycemic effect of glycyrrhizic acid, a natural non-carbohydrate sweetener, on streptozotocin-induced diabetic mice. Food Funct..

[168] Thu V.T., Yen N.T.H., Ly N.T.H. (2021). Liquiritin from Radix Glycyrrhizae Protects Cardiac Mitochondria from Hypoxia/Reoxygenation Damage. J. Anal. Methods Chem..

[169] Kwak H.G., Lim H.B. (2014). Inhibitory effects of Cnidium monnieri fruit extract on pulmonary inflammation in mice induced by cigarette smoke condensate and lipopolysaccharide. Chin. J. Nat. Med..

[170] Basnet P., Yasuda I., Kumagai N., Tohda C., Nojima H., Kuraishi Y., Komatsu K. (2001). Inhibition of itch-scratch response by fruits of *Cnidium monnieri* in mice. Biol. Pharm. Bull..

[171] Hao Y., Liu Y. (2016). Osthole Alleviates Bleomycin-Induced Pulmonary Fibrosis via Modulating Angiotensin-Converting Enzyme 2/Angiotensin-(1-7) Axis and Decreasing Inflammation Responses in Rats. Biol. Pharm. Bull..

[172] Che Y., Li J., Li Z., Li J., Wang S., Yan Y., Zou K., Zou L. (2018). Osthole enhances antitumor activity and irradiation sensitivity of cervical cancer cells by suppressing ATM/NF-κB signaling. Oncol. Rep..

[173] Wang L., Yang L., Lu Y., Chen Y., Liu T., Peng Y., Zhou Y., Cao Y., Bi Z., Liu T. (2016). Osthole Induces Cell Cycle Arrest and Inhibits Migration and Invasion via PTEN/Akt Pathways in Osteosarcoma. Cell. Physiol. Biochem..

[174] Chou S.Y., Hsu C.S., Wang K.T., Wang M.C., Wang C.C. (2007). Antitumor effects of Osthol from *Cnidium monnieri*: An in vitro and in vivo study. Phytother. Res..

[175] Liang H.J., Suk F.M., Wang C.K., Hung L.F., Liu D.Z., Chen N.Q., Chen Y.C., Chang C.C., Liang Y.C. (2009). Osthole, a potential antidiabetic agent, alleviates hyperglycemia in db/db mice. Chem. Biol. Interact..

[176] Lee W.H., Lin R.J., Lin S.Y., Chen Y.C., Lin H.M., Liang Y.C. (2011). Osthole enhances glucose uptake through activation of AMP-activated protein kinase in skeletal muscle cells. J. Agric. Food Chem..

[177] Hu J., Liu R., Yang Z., Pan X., Li Y., Gong Y., Guo D. (2023). Praeruptorin A inhibits the activation of NF-κB pathway and the expressions of inflammatory factors in poly (I:C)-induced RAW264.7 cells. Chem. Biol. Drug Des..

[178] Yu C.L., Yu Y.L., Yang S.F., Hsu C.E., Lin C.L., Hsieh Y.H., Chiou H.L. (2021). Praeruptorin A reduces metastasis of human hepatocellular carcinoma cells by targeting ERK/MMP1 signaling pathway. Environ. Toxicol..

[179] Qian L., Xu Z., Zhang W., Wilson B., Hong J.S., Flood P.M. (2007). Sinomenine, a natural dextrorotatory morphinan analog, is anti-inflammatory and neuroprotective through inhibition of microglial NADPH oxidase. J. Neuroinflamm..

[180] Gao T., Hao J., Wiesenfeld-Hallin Z., Wang D.Q., Xu X.J. (2013). Analgesic effect of sinomenine in rodents after inflammation and nerve injury. Eur. J. Pharmacol..

[181] Su M.X., Song M., Sun D.Z., Zhao H., Gu X., Zhu L., Zhan X.L., Xu Z.N., Wen A.D., Hang T.J. (2012). Determination of sinomenine sustained-release capsules in healthy Chinese volunteers by liquid chromatography-tandem mass spectrometry. J. Chromatogr. B Anal. Technol. Biomed. Life Sci..

[182] Chen Y., Zhang L., Lu X., Wu K., Zeng J., Gao Y., Shi Q., Wang X., Chang L.S., He D. (2014). Sinomenine reverses multidrug resistance in bladder cancer cells via P-glycoprotein-dependent and independent manners. Pharmazie.

[183] Qiu J., Wang M., Zhang J., Cai Q., Lu D., Li Y., Dong Y., Zhao T., Chen H. (2016). The neuroprotection of Sinomenine against ischemic stroke in mice by suppressing NLRP3 inflammasome via AMPK signaling. Int. Immunopharmacol..

[184] Shukla S.M., Sharma S.K. (2011). Sinomenine inhibits microglial activation by Aβ and confers neuroprotection. J. Neuroinflamm..

[185] Cardoso L.P., de Sousa S.O., Gusson-Zanetoni J.P., de Melo Moreira Silva L.L., Frigieri B.M., Henrique T., Tajara E.H., Oliani S.M., Rodrigues-Lisoni F.C. (2023). Piperine Reduces Neoplastic Progression in Cervical Cancer Cells by Downregulating the Cyclooxygenase 2 Pathway. Pharmaceuticals.

[186] McNeil B.D. (2021). MRGPRX2 and Adverse Drug Reactions. Front. Immunol..

[187] Dai Z., Liao X., Wieland L.S., Hu J., Wang Y., Kim T.H., Liu J.P., Zhan S., Robinson N. (2022). Cochrane systematic reviews on traditional Chinese medicine: What matters-the quantity or quality of evidence?. Phytomedicine.

[188] You L., Liang K., An R., Wang X. (2022). The path towards FDA approval: A challenging journey for traditional Chinese medicine. Pharmacol. Res..

[189] Li X., Thai S., Lu W., Sun S., Tang H., Zhai S., Wang T. (2018). Traditional Chinese medicine and drug-induced anaphylaxis: Data from the Beijing pharmacovigilance database. Int. J. Clin. Pharm..

[190] GTEx Consortium (2013). The Genotype-Tissue Expression (GTEx) project. Nat. Genet..

[191] Subramanian H., Gupta K., Ali H. (2016). Roles of Mas-related G protein-coupled receptor X2 on mast cell-mediated host defense, pseudoallergic drug reactions, and chronic inflammatory diseases. J. Allergy Clin. Immunol..

[192] Azimi E., Reddy V.B., Shade K.C., Anthony R.M., Talbot S., Pereira P.J.S., Lerner E.A. (2016). Dual action of neurokinin-1 antagonists on Mas-related GPCRs. JCI Insight.

[193] Peng X., Knapp B.I., Bidlack J.M., Neumeyer J.L. (2007). Pharmacological properties of bivalent ligands containing butorphan linked to nalbuphine, naltrexone, and naloxone at mu, delta, and kappa opioid receptors. J. Med. Chem..

[194] Rehrauer K.J., Cunningham C.W. (2023). IUPHAR Review—Bivalent and bifunctional opioid receptor ligands as novel analgesics. Pharmacol. Res..

[195] Cunningham C.W., Elballa W.M., Vold S.U. (2019). Bifunctional opioid receptor ligands as novel analgesics. Neuropharmacology.

